# Potential antifungal targets based on histones post-translational modifications against invasive aspergillosis

**DOI:** 10.3389/fmicb.2022.980615

**Published:** 2022-08-09

**Authors:** Yiman Li, Zhihui Song, Ente Wang, Liming Dong, Jie Bai, Dong Wang, Jinyan Zhu, Chao Zhang

**Affiliations:** ^1^Department of Pharmacy, Beijing Tongren Hospital, Capital Medical University, Beijing, China; ^2^Department of Hematology, Beijing Tongren Hospital, Capital Medical University, Beijing, China

**Keywords:** invasive aspergillosis, histone post-translational modifications, histone acetylation, histone deacetylation, histone methylation

## Abstract

As a primary cause of death in patients with hematological malignancies and transplant recipients, invasive aspergillosis (IA) is a condition that warrants attention. IA infections have been increasing, which remains a significant cause of morbidity and mortality in immunocompromised patients. During the past decade, antifungal drug resistance has emerged, which is especially concerning for management given the limited options for treating azole-resistant infections and the possibility of failure of prophylaxis in those high-risk patients. Histone posttranslational modifications (HPTMs), mainly including acetylation, methylation, ubiquitination and phosphorylation, are crucial epigenetic mechanisms regulating various biological events, which could modify the conformation of histone and influence chromatin-associated nuclear processes to regulate development, cellular responsiveness, and biological phenotype without affecting the underlying genetic sequence. In recent years, fungi have become important model organisms for studying epigenetic regulation. HPTMs involves in growth and development, secondary metabolite biosynthesis and virulence in *Aspergillus*. This review mainly aims at summarizing the acetylation, deacetylation, methylation, demethylation, and sumoylation of histones in IA and connect this knowledge to possible HPTMs-based antifungal drugs. We hope this research could provide a reference for exploring new drug targets and developing low-toxic and high-efficiency antifungal strategies.

## Introduction

The incidence of invasive fungal disease (IFD) has increased dramatically in recent decades, which is related to the clinical application of broad-spectrum antibiotics, immunosuppressive drugs or antitumor drugs, the development of bone marrow transplantation or other organ transplantation, and the application of long-term and high-dose glucocorticoids, seriously threatens the life of high-risk patients (Enoch et al., 2017; von Lilienfeld-Toal et al., 2019). Aspergillosis invasive (IA) is the second most prevalent invasive fungal illness, and its prevalence continues to rise. IA is a deep fungal infection caused by *Aspergillus* infection, which mostly occurs in immunocompromised patients and results in a high case-fatality rate. The pathogenic *Aspergillus* mainly include *A. fumigatus*, *A. flavus*, *A. niger*, *A. terreus* and *A. nidulans*. *A. fumigatus* is the primary cause of aspergillosis, accounting for 80–90% of IA which has a significant death rate (>60%) in immunocompromised patients without proper treatment (Sugui et al., 2014; Cadena et al., 2021). *A. flavus*, which also causes aspergillosis disorders or liver cancer in humans and animals brought on by eating tainted food, is the second most common *Aspergillus* pathogen in immunocompromised patients (Lohmar et al., 2019). *A. nidulans* is a common cause of IA in patients with chronic granulomatous disease (CGD), who have a higher mortality rate, although it seldom causes IA in immunocompromised individuals (Gresnigt et al., 2018). At present, there are few types of low-toxic and high-efficiency antifungal drugs available in clinical practice. The emergence of drug-resistant strains, especially the increasing number of deep drug-resistant fungal infections, has become a worldwide problem that urgently needs to be solved. The pharmaceutical and academic sectors have made the development of novel antifungal medicines capable of addressing this issue a key priority, which possesses a potentially broad spectrum of activity and minimum toxicity (Mani Chandrika and Sharma, 2020).

**FIGURE 1:**
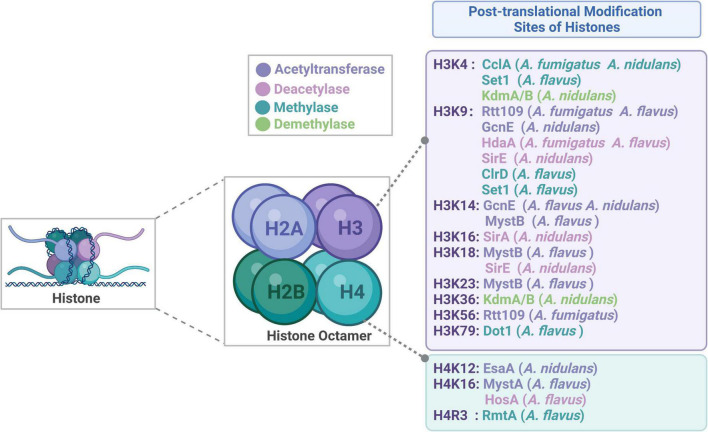
Structure of histone and the sites of histone post-translational modifications.

DNA is bound up with chromatin in the nuclei of eukaryotic cells. The fundamental functional unit of chromatin, the nucleosome, is made up of a histone octamer composed of one H2A-H2B tetramer and two H3-H4 dimers, which is surrounded by 146–147 base pairs of DNA (Figure 1; Jenuwein and Allis, 2001; Zhang et al., 2021b; Lin et al., 2022). The amino residues at the N-terminal and C-terminal tails of histones could be post-translationally modified, such as acetylation, methylation, phosphorylation and ubiquitination, which could alter the electronic charge and structure of these histone tails, thereby altering the chromatin state and subsequent gene expression. It is generally known that histone post-translational modifications (HPTMs) in eukaryotic cells provide a crucial link between biological processes and chromatin-based regulation (Nie et al., 2016; Saavedra et al., 2018). HPTMs could not only control the silencing, activation, expression of various genes but also modulate DNA repairment (Garzon-Porras et al., 2022). The changes of histone modification in tumor cells have attracted a lot of attention, and the related drugs have been used in the clinical treatment of tumors with higher curative effects and lower side effects (Xu et al., 2021; Lopez et al., 2022). Research on HPTMs is currently concentrated on metabolism, tumor treatment, and other areas, there is currently relatively limited research on microbial HPTMs, particularly with regard to microbial pathogenicity. Recent advances in pathogenic microorganisms indicate that fungal pathogens could modify their histone modification status to control their growth, development, virulence features, and drug resistance. Recent studies revealed that histone acetyltransferases (HATs) and histone deacetylases (HDACs) play crucial roles in the development, virulence, drug resistance and stress responses of *Candida albicans* (*C. albicans*) (Kim et al., 2015). In addition, HPTMs may work with well-known transcription factors in the signaling pathways to contribute to the morphological change in *C. albicans* (Kim et al., 2015; Kim J. et al., 2018; Su et al., 2020a). Studies have shown that HPTMs are involved in multiple physiological processes in *Aspergillus*, which can be developed as potential targets for novel antifungal drugs in the future.

**FIGURE 2:**
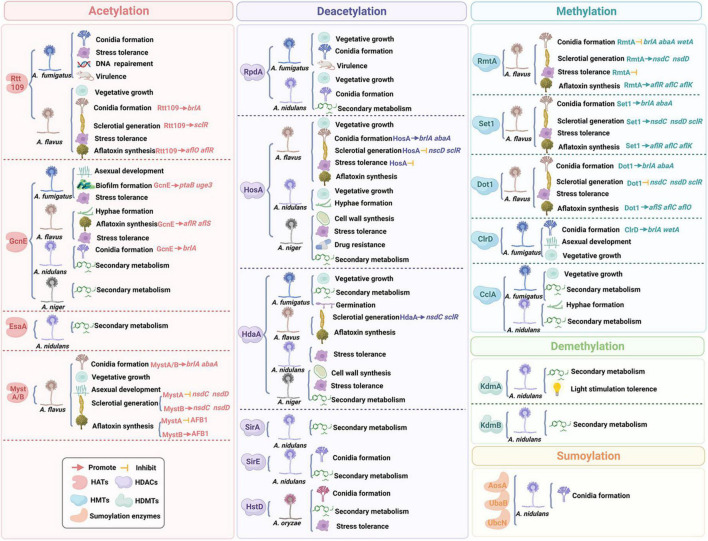
A schematic representation of the relationship between histone post-translational modifications and fungal growth and virulence.

In this review, we discussed the composition of histones and widespread distribution of HPTMs in *Aspergillus* (Figure 1), especially in the *A. fumigatus*, *A. flavus*, *A. nidulans*, *A. niger*, and *A. oryzae*. Then, we highlighted recent examples to illustrate the role of HPTMs, mainly including HATs, HDACs, histone methylases (HMTs), histone demethylases (HDMTs) and sumoylation enzymes in *Aspergillus* (Figure 2). Finally, we reviewed the HPTMs in yeast and *Aspergillus* (Table 1). In addition, we summarized the drugs or compounds which possess anti-*Aspergillus* activity targeting HPTMs, mainly based on HDACs (Table 2), in order to provide alternative targets for new antifungal drug development and provide a potential resource for sensible antifungal usage of antifungals in clinical practice.

**TABLE 1:** Histones post-translations modifications in yeast and *Aspergillus.*

HPTMs Classes	HPTMs Sites				References
	Yeast		*Aspergillus*		
Acetylation	Rtt109	*S. cerevisiae*	Rtt109	*A. fumigatus* *A. flavus*	Cheng et al., 2016
	Gcn5	*S. cerevisiae*	GcnE	*A. nidulans* *A. fumigatus* *A. flavus* *A. niger*	Abu et al., 2016
	Esa1	*S. cerevisiae*	EsaA	*A. nidulans*	Smith et al., 1998
	Sas2	*S. cerevisiae*	MystA	*A. flavus*	Chen et al., 2022
	Sas3	*S. cerevisiae*	MystB	*A. flavus*	Chen et al., 2022
Deacetylation	Rpd3	*S. cerevisiae*	RpdA	*A. nidulans* *A. fumigatus*	Tribus et al., 2010
	Hos2	*S. cerevisiae*	HosA	*A. nidulans* *A. flavus* *A. niger*	Pfaller et al., 2009
	Hda1	*S. cerevisiae*	HdaA	*A. nidulans* *A. fumigatus* *A. flavus* *A. niger*	Tribus et al., 2005
	Sir2	*S. cerevisiae*	SirA	*A. nidulans*	Shimizu et al., 2012
	Hst3	*S. cerevisiae*	SirE	*A. nidulans*	Frye, 2000
	Hst4	*S. cerevisiae*	HstD	*A. oryzae*	Yang et al., 2008
Methylation	Hmt1	*S. cerevisiae*	RmtA	*A. flavus*	Mcbride et al., 2000
	Set1	*S. cerevisiae*	Set1	*A. flavus*	Briggs et al., 2001
	Dot1	*S. cerevisiae*	Dot1	*A. flavus*	Valencia-Sanchez et al., 2021
	Clr4	*Schizosaccharomyces pombe*	ClrD	*A. fumigatus*	Nakayama et al., 2001
	Bre2	*S. cerevisiae*	CclA	*A. nidulans* *A. fumigatus*	Eissenberg and Shilatifard, 2010
Demethylation	Rph1	*S. cerevisiae*	KdmA	*A. nidulans*	Gacek-Matthews et al., 2015
	Jhd2	*S. cerevisiae*	KdmB	*A. nidulans*	Gacek-Matthews et al., 2016
Sumoylation	Aos1	*S. cerevisiae*	AosA	*A. nidulans*	Johnson et al., 1997
	Uba2	*S. cerevisiae*	UbaB	*A. nidulans*	Johnson et al., 1997
	Ubc9	*S. cerevisiae*	UbcN	*A. nidulans*	Johnson and Blobel, 1997
	Siz1	*S. cerevisiae*	SizA	*A. nidulans*	Johnson and Gupta, 2001

**TABLE 2:** Antifungal activities of compounds against *Aspergillus* spp. based on HPTMs.

Potential antifungal targets	Antifungal compounds	Strains	Antifungal activities (alone)	Combined drugs	Antifungal activities (combined)		FICI	References
					Antifungal compounds	Combined drugs		
HDAC	Trichostatins A	*A. fumigatus* (AF293)	MEC = 0.5 μg/ml	–	–	–	–	Lamoth et al., 2014
		*A. fumigatus* (AF293)	MIC_50_ = 1 μg/ml	–	–	–	–	
		*A. fumigatus* (AF293)	MIC_90_ = 4 μg/ml	–	–	–	–	
		*A. fumigatus* (EMFR-S678P)	MEC = 4 μg/ml	Caspofungin	–	–	FICI < 0.5 Synergy	
		*A. flavus* (*n* = 4)	MEC_50_ = 2 μg/ml	–	–	–	–	Lamoth et al., 2015
		*A. niger* (*n* = 2)	MEC_50_ (8 to > 16 μg/ml)	–	–	–	–	
HDAC	Vorinostat	*A. fumigatus* (AF103)	MIC_100_ ≥ 16 μg/ml	Itraconazole	2 μg/ml	0.5 μg/ml	FICI = 0.31 Synergy	Tu et al., 2020
		*A. fumigatus* (AF103)	MIC_100_ ≥ 16 μg/ml	Voriconazole	2 μg/ml	0.25 μg/ml	FICI = 0.31 Synergy	
		*A. flavus* (AFLA101)	MIC_100_ ≥ 16 μg/ml	Itraconazole	2 μg/ml	0.5 μg/ml	FICI = 0.31 Synergy	
		*A. flavus* (AFLA101)	MIC_100_ ≥ 16 μg/ml	Voriconazole	2 μg/ml	0.25 μg/ml	FICI = 0.31 Synergy	
		*A. fumigatus* (AF104)	SMIC_80_ ≥ 256 μg/ml	Itraconazole	64 μg/ml	64 μg/ml	FICI = 0.25 Synergy	
		*A. fumigatus* (AF104)	SMIC_80_ ≥ 256 μg/ml	Voriconazole	64 μg/ml	64 μg/ml	FICI = 0.25 Synergy	
		*A. flavus* (AFLA4)	SMIC_80_ ≥ 256 μg/ml	Voriconazole	128 μg/ml	64 μg/ml	FICI = 0.38 Synergy	
HDAC	Givinostat	*A. fumigatus* (AF12)	MIC_100_>16 μg/ml	Itraconazole	2 μg/ml	0.25 μg/ml	FICI < 0.5 Synergy	Sun et al., 2017
		*A. fumigatus* (AF12)	MIC_100_>16 μg/ml	Voriconazole	4 μg/ml	0.125 μg/ml	FICI < 0.5 Synergy	
		*A. fumigatus* (AF12)	MIC_100_>16 μg/ml	Posaconazole	2 μg/ml	0.125 μg/ml	FICI < 0.5 Synergy	
		*A. flavus* (AFL10)	MIC_100_>16 μg/ml	Voriconazole	4 μg/ml	0.25 μg/ml	FICI < 0.5 Synergy	
		*A. flavus* (AFL10)	MIC_100_>16 μg/ml	Posaconazole	4 μg/ml	0.125 μg/ml	FICI < 0.5 Synergy	
HDAC	MGCD290	*A. fumigatus* (*n* = 3)	MIC_50_ (0.5–8 μg/ml)	Fluconazole	–	–	FICI < 0.5 Synergy	Pfaller et al., 2009
		*A. flavus* (*n* = 2)	MIC_50_ (8- > 16 μg/ml)	Fluconazole	–	–	FICI < 0.5 Synergy	

MEC, minimal effective concentration; MIC, minimal inhibitory concentration; SMIC, sessile minimal inhibitory concentration; FICI, fractional inhibitory concentration index; “–” refers to not available.

## Histone acetyltransferases

The post-translational modification of histone acetylation was the first known chromatin modification mechanism and has been extensively reported in recent years (Buuh et al., 2017; Chen et al., 2022). The acetylation of histones is a dynamic and reversible post-translational alteration of proteins, which is involved in the regulation of gene transcription, protein stability and cellular metabolism (Zhang et al., 2018). Histone acetylation modification was coordinated by HATs and HDACs on the N-terminal of histone lysine residues (Zhang et al., 2021a; Chen et al., 2022). HAT could covalently attach an acetyl group (usually provided by acetyl-CoA) to the ε-amino side chain of lysine. The positively charged lysine could be neutralized by the negatively charged acetyl group, thereby reducing the interaction between histones and DNA, forming a looser chromatin structure named euchromatin, which is conducive to gene transcription (Lennartsson and Ekwall, 2009). Recent research has demonstrated that HATs regulate *Aspergillus* growth, response to environmental stress, and pathogenicity (Zhang et al., 2021a).

### Rtt109

Regulators of Ty1 translocation109 (Rtt109) is a unique HAT in fungi and the sequence is conservative in most filamentous fungi (Lopes da Rosa et al., 2013). Rtt109’s core catalytic domain has a similar three-dimensional structure to that of the mammalian HAT p300/CBP, but their catalytic mechanisms are distinct (Zhang et al., 2021a). Rtt109 is reported to acetylate histone H3 on K9, K27, and K56 (Zhang et al., 2021a). Histone molecular chaperone Asf1 integrates with H3, and then H3K56 is acetylated by Rtt109. Vps75 is a member belongs to the family of the nucleosome assembly proteins (NAP-1). It is another molecular chaperone related to Rtt109 which was first found in *Saccharomyces cerevisiae* (*S. cerevisiae*). Vps75 and Rtt109 could interact to produce the Rtt109-Vps75 complex with a special functional domain, which affects the acetylation level of H3K56 (Tang et al., 2008). The involvement of Rtt109 in pathogenic fungi has drawn growing attention due to its implications in the development of novel antifungals. It is hypothesized that Rtt109 acetylation may have a role in maintaining genomic stability, transcriptional control, and DNA damage repair(Zhang et al., 2021a).

In *A. fumigatus*, Rtt109-Asf1-H3-H4 complex functions as an integral substrate unit which is essential for H3K9 and H3K56 acetylation (Zhang et al., 2018). Rtt109 has histone acetyltransferase activity both *in vitro* and *in vivo* (Zhang et al., 2021a). Recent research has confirmed that Rtt109 controls the regulation of conidiation, oxidative stress, cellular metabolism and DNA damage repair in *A. fumigatus* (Zhang et al., 2021a). Comparing the mutant strain to the wild-type and complemented strains, the Δ*rtt109* mutant drastically reduced conidia production and colony growth (Yang G. et al., 2019). Gene Ontology (GO) enrichment analysis showed that in Δ*rtt109* mutant most genes were upregulated and the majority of upregulated genes were involved in oxidation-reduction processes, transmembrane transport, carbohydrate metabolism and amino acid metabolism, while the expression of DNA damage genes was downregulated in the Δ*rtt109* mutant (Kwon et al., 2018). In addition, Rtt109 is a crucial part of genotoxic stress tolerance. Rtt109 is critical for the virulence of *A. fumigatus*, survival analysis showed that Δ*rtt109* mutant exhibited a significantly reduced mortality rate.

In *A. flavus*, H3K9 is the acetylation target of Rtt109, which played important role in its full functions. Recent studies have shown that Rtt109 contributes to *A. flavus*’s vegetative growth, conidia formation, sclerotia production, aflatoxin synthesis, and response to environmental stress (Sun et al., 2021). In the Δ*rtt109* mutant, the vegetative growth, conidial formation and sclerotial generation were significantly suppressed in *A. flavus*, and the expression levels of the related gene *brlA* and *sclR* were lower compared to the wild type (WT) and Δ*rtt109* complementary strains. *A. flavus* is the primary producer of aflatoxins, which have been determined to be one of the most harmful and cancer-causing secondary metabolites (SM) (Yang K. et al., 2019). A persistent exposure to aflatoxins may impair the immune response and result in malnutrition, hepatic lesions and even hepatomas (Yang et al., 2018; Yang K. et al., 2019). In Δ*rtt109* mutant, the production of aflatoxin decreased significantly, the expression levels of aflatoxin-specific regulatory genes *aflO* and *aflR* had quite low levels of expression. Moreover, Rtt109 is critical in responding to cell wall stress, oxidative damage, osmotic stress and particularly genotoxic stress in *A. flavus*. The research results of Rtt109 provide evidence for the role of acetylation in the regulation of the life process of *A. flavus* and facilitate the prevention and control of *A. flavus*.

### GcnE

General control non-derepressible E (GcnE) (Gcn5) is a kind of HATs that could release histones from chromatin to initiate transcriptional activation. The SAGA (Spt-Ada-GcnE) complex consists of the acetyltransferase GcnE, two transcriptional adapters (Ada2 and Ada3), and proteins such as Spt3, which is conserved in eukaryotic cells and could participate in the regulation of a variety of gene transcription (Koutelou et al., 2010). GcnE has been linked to the growth, development, stress tolerance and virulence in *Candida albicans*, *Candida glabrata*, *Cryptococcus neoformans* and other fungal species (O”Meara et al., 2010; Peng et al., 2015). In *Aspergillus*, research have shown that GcnE is essential for fungal growth and SM synthesis.

In *A. fumigatus*, GcnE has been shown in studies to play a role in the regulation of asexual development, stress response and biofilm formation (Nossmann et al., 2018; Lin et al., 2020). The Δ*gcnE* mutant was defected in asexual development with abnormal phialide formation. GcnE deficiency increased sensitivity to the cell wall perturbing agents (Congo Red, SDS) and oxidative stress inducing agents (H_2_O_2_, menadione), whereas the Δ*gcnE* mutant showed enhanced tolerance to osmotic stress inducing agents (LiCl) in comparison to the wild-type and complemented strains. Moreover, GcnE could control conidiogenesis, germination and biofilm formation of *A. fumigatus* and the biofilm formation was regulated by controlling the expression of *ptaB* and *uge3* (Nossmann et al., 2018; Lin et al., 2020). GcnE does not seem to control virulence in an invasive *A. fumigatus* which is neutropenic.

In *A. flavus*, the acetylation target of GcnE is H3K14. GcnE plays an indispensable role in growth rate, sclerotial formation, morphological development, aflatoxin biosynthesis, stress responses and pathogenicity (Lan et al., 2016; Cai et al., 2018). The Δ*gcnE* mutant produced less mycelia and fewer branches at the mycelial tips than the WT and complementing strains according to microscopic analyses. Moreover, the cell surface hydrophobicity of the Δ*gcnE* mutant was decreased, which may be related to the inhibited production of aerial hyphae. Δ*GcnE* mutant was unable to produce the aflatoxin B1 (AFB1) and AFB2, the aflatoxin-specific regulatory genes (*aflR*, *aflS*) were lower than in the other two strains. In addition, GcnE is involved in responses to cell wall (calcofluor white, Congo red) and genotoxic stresses (methyl methanesulfonate), but not to hyperosmotic stresses (NaCl, KCl) or oxidative stresses (H_2_O_2_). GcnE may be a potential target for preventing *A. flavus* infections.

In *A. nidulans*, GcnE function was found to be required for the acetylation of histone H3K9/K14 (Qi et al., 2018). Conidiophore development was not observed in the Δ*gcnE* mutant, but it was restored when the *gcnE* deletion was complementated (Cánovas et al., 2014). Transcriptome analysis revealed that GcnE was involved in the regulation of conidiation and secondary metabolism genes, analysis of the gene expression revealed that the lack of conidiation originated in a complete absence of *brlA* expression in the Δ*gcnE* strain (Cánovas et al., 2014). Deletion of the *gcnE* gene decreased the production of selenic acid, sterigmatocystin, penicillin and terrequinone (Nuetzmann et al., 2011).

In *A. niger*, GcnE is associated with the production of multiple polyketide metabolites production. Studies have reported that 12 polyketide secondary metabolites were produced when the epigenetic regulator gene *gcnE* was deleted in *A. niger* (Wang et al., 2018; Li X. et al., 2021).

### MYSTs

The MYST (Moz, YBF2, Sas2p, Tip) proteins are involved in a variety of biological processes, including gene regulation, DNA repair, cell-cycle regulation, and development. They are largely conserved from yeast to humans (Su et al., 2020a). MYST proteins constitute the largest HATs family and are named for the founding members MOZ, Ybf2/Sas3, Sas2, and TIP60 (Luan et al., 2016). In *S. cerevisiae*, Mysts contain three members including Esa1, Sas2, and Sas3, which function in DNA replication regulation and transcriptional silencing (Sapountzi and Côté, 2011).

EsaA (Esa1, Essential SAS family acetyltransferase) is the human homolog of Tip60, which mainly acetylates histone H4 lysine 5, 8, 12, 14. In *A. nidulans*, esaA serves a new involvement in the activation of four SM gene clusters via histone 4 lysine 12 (H4K12) acetylation (sterigmatocystin, penicillin, terrequinone, and orsellinic acid). Acetylation of SM gene promoters is increased by *esaA* overexpression. H4K12 concentrations might serve as a special indicator of relative production potential, particularly for SMs (Smith et al., 1998; Soukup et al., 2012).

In *A. flavus*, MystA (Sas2 orthologs) and MystB (Sas3 orthologs) are two traditional non-essential Myst enzymes found in nucleus and cytoplasm (Chen et al., 2022). MystA could acetylate H4K16, while MystB acetylates H3K14, H3K18, and H3K23. In the aspects of conidia formation, the regulatory genes of conidia formation (*brlA* and *abaA*) were significantly decreased in Δ*MystB* and Δ*MystA*/*MystB*. In *A. flavus*, MystA and MystB are necessary for vegetative development and the formation of asexual spores, and MysB has a significant function (Chen et al., 2022). In respect of sclerotia formation, MystA and MystB play opposite roles in the sclerotia regulation. Sclerotia is a dormant body interwoven with hyphae, which can resist adverse environments when fungi encounter environmental stress. When the environment is suitable, the sclerotia could germinate to produce new hyphae or form new propagules. Two essential regulatory genes (*nsdC* and *nsdD*), required for the production of sclerotia, were both markedly downregulated in the Δ*MystB* and Δ*MystA*/*MystB* but significantly increased in the Δ*MystA* (Chen et al., 2022). The research indicated that MystA performed a detrimental function in sclerotium development, but MystB was essential in *A. flavus* (Chen et al., 2022). In the aspect of aflatoxins biosynthesis, MystA and MystB played distinct but significant roles in the biosynthesis of AFB1, and MystB played an essential role in the regulation of aflatoxin (Chen et al., 2022).

## Histone deacetylases

Histone deacetylation is the most widely studied among fungal epigenetic modifications (Bauer and Graessle, 2021). HDACs are important in eukaryotic cells which play an essential role in gene expression, transcription and post-transcriptional protein modification by reducing the acetylation level of histones. HDACs are currently divided into three classes in *Aspergillus*: (1) class I enzymes RpdA and HosA, two enzymes of RPD3-type; (2) class II HDACs HdaA and HosB, two enzymes of HDA1-type; (3) several members of the sirtuins class (Brosch et al., 2008; Tribus et al., 2010).

Histone deacetylases are important targets in tumor therapy, interfering with the acetylation pattern may provide a promising tool to attack diseases (Kim J. et al., 2018). The overexpression of HDACs in cancer cells breaks the acetylation balance of the original gene in transcription and expression, leading to tumorigenesis. HDAC inhibitor (HDACi) can inhibit the activity of HDACs in tumor cells, increase the degree of histone acetylation in tumor cells, re-activate the inhibited tumor suppressor genes, and then induce tumor cell differentiation and apoptosis. In recent years, the design and development of new HDACi has become a hot topic in the field of antitumor drugs, which is of great significance for the treatment of tumor diseases. Several HDAC inhibitors have been approved for clinical use. Non-selctive HDACi vorinostat has been approved to treat the cutaneous T-cell lymphoma, panobinostat could repress the metastasis in triple-negative breast cancer (Li and Seto, 2016; Luan et al., 2019).

Fungal HDACs are crucial in the stress signaling pathway of fungal drug resistance through epigenetic regulation, various virulence factors such as adhesion, hyphal formation and biofilm production are also regulated by fungal HDACs (Patrick et al., 2003; Su et al., 2020a). In *C. albicans*, HDAC is related to fungal pathogenicity and virulence, especially in the aspect of drug resistance. Studies have shown that HDAC inhibitors in combination with azoles against drug-resistant *C. albicans* showed synergistic effects *in vitro*, and histone deacetylation modification may be one of the upstream resistant mechanisms of *C. albicans* (Su et al., 2020b). The study of modification in *Aspergillus* will improve our understanding of the pathogenic fungus *Aspergillus* and provide a theoretical foundation for the prevention and management of fungi.

### RpdA

RpdA is an RPD3-type HDAC of *Aspergillus*, it has become more and more evident that RpdA affects numerous biological processes in *Aspergillus*. A conserved fungal-specific C-terminal motif found in RpdA protein is necessary for nucleus localization, enzymatic activity, and viability during axenic development. RpdA is a viable target for new, fungus-focused inhibitors due to its fungal-specific motif.

In *A. fumigatus*, studies have shown that RpdA was related to vegetative growth and sporulation. The HDAC inhibitor Trichostatin A (TSA) decreased the growth of *A. fumigatus*. Earlier studies showed that single or even combined deletion of the three remaining HDACs (HdaA, HosA, and HosB) did not result in comparable defects in germination, growth or development (Shwab et al., 2007), it is reasonable to speculate that the main effect of TSA therapy is the suppression of RpdA activity. Furthermore, researches have demonstrated that *A. fumigatus* became avirulent in a mouse model of pulmonary aspergillosis due to downregulation of *RpdA* (Bauer et al., 2016, 2019).

In *A. nidulans*, RpdA is in charge of deacetylating H3 and H4. RpdA played an essential role in fungal development, the suppression of *rpdA* resulted in the loss of viability, growth and sporulation of *A. nidulans* (Tribus et al., 2010). In addition, RpdA could participate in SM biosynthesis. A large-scale metabolomics study of an *A. nidulans*Δ*rpdA* mutant showed that RpdA was involved in positively and negatively regulating various products, downregulation of several known metabolites, primarily sterigmatocystin and the emericellamides, was caused by RpdA disturbance (Albright et al., 2015).

### HosA

In *A. flavus*, H4K16 is the deacetylated target of HosA. Through functional analysis, it was found that the class I deacetylase HosA is involved in the growth, spore formation, sclerotium production, oxidative stress response and toxin production of *A. flavus*. When compared to WT and complementing Δ*hosA* strains, the Δ*hosA* mutant grew more slowly and produced much less conidia. HosA is necessary for the fungal vegetative growth and asexual development. In comparison to the WT and complimentary strains, the *brlA* and *abaA* essential regulatory genes, which are necessary for the activation of conidiation, were both dramatically downregulated in the Δ*hosA* mutant. Alternative reproduction and survival mechanisms known as sclerotia are used to adapt to unfavorable environmental conditions, while HosA played a negative role in sclerotial formation. *NscD* and *sclR* are transcription regulators encoding sclerotia formation, both gene expressions were increased substantially in the Δ*hosA* strains. In addition, HosA has detrimental effects on the oxidative stress response, and Δ*hos*A strain exhibited increased tolerance to medicines that generate free radicals. HosA is also involved in aflatoxin biosynthesis, in comparison to the WT strain, the Δ*hos*A strain produced less AFB1(Lan et al., 2019).

In *A. nidulans*, HosA is important for the growth and sporulation of colonies, deletion of *HosA* resulted in reduced growth and discoloration of hyphae and medium. In addition, deletion of *HosA* has no effect on *A. nidulans*’s susceptibility to the selected antifungal medications (voriconazole, fluconazole, amphotericin B, and caspofungin) (Pidroni et al., 2018).

In *A. niger*, HosA is associated with phenotypic change, stress resistance and SM biosynthesis. HosA is essential for stress tolerance and drug resistance, the Δ*hosA* strains showed significant defects in response to heat (42°C), oxidative stress (H_2_O_2_), osmotic stress (NaCl) and cell wall stress (calcofluor white). In addition, HosA could affect cell wall synthesis, the expression of genes related to cell wall synthesis was downregulated in Δ*hosA* strains. In the aspect of SM biosynthesis, the synthesis of fumonisin B1 and B2 was reduced (fold change ≥ 2) compared to that of the WT strains in the Δ*hosA* mutant (Li et al., 2019a).

### HdaA

In *A. fumigatus*, HdaA deacetylates H3K9. HdaA is necessary for germination and vegetative growth and is involved in the regulation of SM production. Studies have shown that Δ*hdaA* strains were aberrant in SM production and the virulence factor gliotoxin was repressed. Furthermore, HdaA functioned as a positive regulator of germination and radial growth. Nonetheless, HdaA was to be independent of virulence properties and oxidative stress (Lee et al., 2009).

In *A. flavus*, H3K9 might be the deacetylation target of HdaA. Studies revealed that sclerotia production in the Δ*hdaA* mutant strain reduced dramatically, whereas, the complemented Δ*hdaA.C* strain restored the deficiency. The expression of sclerotia formation-related genes *nsdC* and *sclR* were decreased substantially in the Δ*hdaA* strain. In addition, HdaA was involved in aflatoxin biosynthesis, and the Δ*hdaA* strain produced significantly less AFB1 than the WT strain (Lan et al., 2019).

In *A. nidulans*, HdaA is involved in the control of enzymes that are crucial for the cellular antioxidant response, Δ*hdaA* strains showed a noteble reduction of growth under various oxidative stress settings (Tribus et al., 2005).

In *A. niger*, HdaA exhibited a modest influence on stress tolerance, the Δ*hdaA* strain was slightly sensitive to H_2_O_2_ and heat. Eight genes involved in the formation of cell walls showed downregulated expression in the Δ*hdaA* strain. HdaA played a role in SM biosynthesis. In Δ*hdaA* mutants, the well-known secondary metabolites fumonisin and kojic acid were reduced in the production. Consequently, HDACs could offer new strategies for the regulation of SM synthesis (Li et al., 2019a).

### Sirtuins

Sirtuins (Silent information regulator 2 protein) are Class III HDACs, requiring NAD^+^ for histone deacetylation and participating in multiple biological regulation processes (Imai and Armstrong, 2000; Landry et al., 2000).

Sirtuin A (SirA) is a widely distributed NAD^+^-dependent HDAC with sequence homology to the silent information regulator (Sir2) protein from *S. cerevisiae*, which could convert euchromatin into heterochromatin and silence gene expression (Brosch et al., 2008). In *A. nidulans*, SirA could deacetylate the acetylated 16th lysine residue on histone H4. SirA is a transcriptional repressor of SM synthesis, which could suppress the production of austinol, dehydroaustinol, and sterigmatocystin (Itoh et al., 2017b).

Sirtuin E (SirE) is the Class Ic sirtuin of *A. nidulans* and its deacetylation sites including H3K9, H3K18, and H3K56. Studies have revealed that SirE was involved in autolysis, conidia development and fungal metabolism in *A. nidulans*. The Δ*sirE* strain showed reduced mycelial autolysis and defective conidia development. In addition, the expressions of corresponding regulatory genes were also decreased. Moreover, SirE was the global primary and secondary metabolic regulator during the cell growth cycle (Itoh et al., 2017a).

HstD, the homolog of SirE in *A. nidulans*, is a Class Ic sirtuin in *A. oryzae*. LaeA is a global regulator of secondary metabolism and cell development of filamentous fungi. HstD is the upstream of LeaA which could involve in the regulation of conidial information, SM biosynthesis and stress tolerance, the Δ*hstD* strain was sensitive to the osmotic stress (Kawauchi et al., 2013; Kawauchi and Iwashita, 2014).

## Histone methylases

Histone methylation, which adds methyl groups to the lysine (K) and arginine (R) residues of histone H3 and H4, is one of the most essential post-transcriptional modifications (Gong and Miller, 2017; Li Y. et al., 2021). HMTs are mainly responsible for the methylation of the basic amino acid lysine on histones, adding one to three methyl groups to ε-amine groups to form monomethyl, dimethyl, or trimethyl lysine to act as the active or repressive marks of gene expression (Freitag, 2017; Zhang et al., 2021b). Histone methylation is inextricably linked to gene expression control, epigenetic disorders are an effective target for fungal treatment interventions.

### RmtA

Protein arginine methyltransferases (PRMTs) are capable of catalyzing posttranslational arginine methylation. RmtA is the homolog of human PRMT1, which has intrinsic histone methyltransferase activity and methylates histone H4 at arginine 3 (H4R3) (Li et al., 2017). In *A. flavus*, studies have shown that RmtA was a positive regulator of sclerotia formation and aflatoxin development (Satterlee et al., 2019). In Δ*rmtA* mutants, there were fewer sclerotia formed compared to WT, the expression of related genes, *nsdC* and *nsdD*, were downregulated. RmtA gene played an important role in AFB1 biosynthesis, when compared to WT, Δ*rmtA* mutants displayed lower expression levels of the AFB1 synthesis-regulating gene *aflR* and the AFB1 biosynthesis genes *aflC*, and *aflK* (Li et al., 2017). Nevertheless, RmtA was a negative regulator of conidia development and oxidative stress tolerance. The expression levels of conidia development regulatory genes *brlA*, *abaA*, and *wetA* were significantly improved in the Δ*rmtA* mutants (Li et al., 2017), the Δ*rmtA* mutants presented an increased tolerance to oxidative stress compared to WT (Satterlee et al., 2016; Li et al., 2017).

### Set1

In *A. flavus*, Set1 controls H3K4 and H3K9 dimethylation, and H3K4 trimethylation, which provides substantial evidence for identifying the biological activities of histone methyltransferase in pathogenic fungi. Studies revealed that Set1 was involved in the regulation of growth and pathogenicity of *A. flavus*. Set1 positively regulates colony formation and increases conidiation via an *abaA* and *brlA* mediated mechanism. Furthermore, Set1 controls the production of sclerotia via conventional sclerotia formation regulators, the expression levels of the major sclerotia formation regulators (*nsdC*, *nsdD* and *sclR*) were significantly reduced in the Δ*Set1* strain. In the respect of aflatoxin synthesis, Set1 positively participates in the biological synthesis of AFB1. In the traditional aflatoxin production pathway, the expression levels of major transcriptional factor genes (*aflR* and *aflS*) as well as the enzyme genes (*aflC*, *aflD*, *aflK*, and *aflO*) were substantially down-regulated in Δ*Set1* strain compared to WT. In addition, Set1 is also involved in environmental stress regulation including osmotic stress (H_2_O_2_), oxidative stress (NaCl, KCl) and plasma membrane stress (SDS). This might offer a genetic target for combating the detrimental effects of the activity of *A. flavus* (Li et al., 2017; Liu et al., 2020).

### Dot1

Dot1 histone methyltransferase, which targets H3K79, is essential for meiotic checkpoint function. The positions and paralogs of Dot1 were notably conserved among *Aspergillus* genomes in terms of their biological roles. In *A. flavus*, Dot1 is 33% identical to yeast Dot1 generally (Ontoso et al., 2013). Studies have indicated that Dot1 was related to aflatoxin production, fungal pathogenicity, and fungal development. Disruption of the H3K79 methyltransferase gene *dot1* reduced *A. flavus* radial growth and conidiation by down-regulating the transcriptional levels of the key transcription factors *brlA* and *abaA*. Dot1 contributes to aflatoxin biosynthesis, in the Δ*dot1* mutant, the AF-regulated gene (*aflS*) and the biosynthetic genes (*aflC*, *aflO*) were transcriptionally repressed. Moreover, AF production was drastically reduced. In addition, Dot1 has a potential role in response to genotoxicity stress, cell wall stress and oxidative stress. However, Dot1 plays a negative role in sclerotial reproduction, the transcription of the associated genes (*nsdC*, *nsdD*, *sclR*) was all up-regulated in the Δ*dot1* mutant (Liang et al., 2017). These studies may present a potential target for new strategies to control *A. flavus*.

### ClrD

In *A. fumigatus*, ClrD was the homolog of Clr4, which is the major enzyme responsible for histone H3K9 methylation in *Schizosaccharomyces pombe*. ClrD is associated with the mono- and trimethylation of H3K9 and participates in normal growth and *brlA*-mediated conidiophore development (Palmer et al., 2008).

### CclA

Bre2 is a crucial component of COMPASS in the SPRY domain protein in *S. cerevisiae*. Analysis of the *A. nidulans* genome revealed the existence of a possible ortholog of Bre2 called CclA, which is responsible for H3K4 dimethylation and trimethylation (Bok et al., 2009).

In *A. fumigatus*, Δ*cclA* strain grew weakly, as seen by diminished radial development. In particular, CclA is responsible for SM suppression. In comparison to WT, the Δ*cclA* mutant produced more than four times as much gliotoxin. A significant rise in the transcription of *gliZ*, the C6 transcription factor necessary for the production of *gli*, was linked to a high level of gliotoxin (Palmer et al., 2013).

In *A. nidulans*, CclA is critical in the regulation of phenotypic change and SMs synthesis. Studies have shown that Δ*cclA* led to a decrease in colony diameter and a defect in hyphal morphology (Giles et al., 2011).

## Other histone posttranslational modifications

In addition to histone acetylation, deacetylation and methylation, there are also many histones post-translational modifications in *Aspergillus* species, such as demethylation, sumoylation, ubiquitination and phosphorylation, which could be involved in the regulation of cell growth, transcriptional regulation and secondary metabolism. In this section, we discussed the effects of histone demethylation and sumoylation on *Aspergillus* growth and pathogenicity.

### Histone demethylases

In *A. nidulans*, KdmA (homolog of yeast Rph1) and KdmB (homolog of yeast Jhd2) are two histone demethylases that function as H3K36me3 demethylase and H3K4me3 demethylase respectively (Gacek-Matthews et al., 2015, 2016). KdmA is essential for the tolerance of detrimental light stimulation, which is associated with cell growth and development. Moreover, KdmA could positively regulate the genes transcribed during SM while negatively regulate the genes involving in energy metabolism and protein production and stability. KdmB plays an important role in transcription, especially in SM biosynthesis. Deletion of KdmB caused the dysregulation of genes involved in SM production (about 50% genes).

### Sumoylation enzymes

Small ubiquitin-like modifier (SUMO) is mainly distributed in the nucleus. Sumoylation is an important post-translational modification which participates in the transcriptional regulation, DNA damage repairment and genome integrity maintenance. Sumoylation is a dynamic and reversible process. SUMO could conjugate to lysine residues in target proteins through an isopeptide linkage with the participation of activating enzyme E1 (AosA/UbaB), conjugating enzyme E2 (UbcN) and ligating enzyme E3 (SizA) (Chang and Yeh, 2020). In *A. nidulans*, potential sumoylation targets including histone modifiers (as the COMPASS complex), metabolic and stress response enzymes. Sumoylation was required for both sexual reproduction and asexual development. Deletion of *aosA*, *ubaB*, and *ubcN* resulted in the decreased asexual spore production and defected ascospores formation. In contrast to E1 and E2, E3 might play a special role in cell development, the *sizA* knockout strain displayed WT-like conidiospore formation and sexual development (Harting et al., 2013).

## Potential antifungal compounds related to histone posttranslational modifications

At present, some HPTM inhibitors are being developed as drugs for the treatment of various human diseases. Some HDAC inhibitors for the treatment of cancer, including vorinostat, romidepsin, belinostat, and panobinostat, have received Food and Drug Administration (FDA) approval (Campbell and Thomas, 2017; Sivaraj et al., 2017; Kim Y. H. et al., 2018; Athira et al., 2021; Shi et al., 2021). Although it has not been widely used in the treatment of fungal infections, the use of existing HPTM inhibitors or the use of these proteins as drug targets for the development of new antifungal compounds with great potential. Although these inhibitors may have side effects and toxicity, they are still effective. HPTM inhibitors can not only be applied to the existing approved inhibitors of “drug reuse,” but also be used for large-scale screening methods to determine new compounds for further application in the treatment of fungal infections (Li et al., 2019b). The antifungal activities of compounds targeted HPTMs were summarized in Table 2, and we mainly summarized the compounds targeted HDAC as below.

Trichostatins A (TSA) is a non-selective HDAC inhibitor that displays different degree of *in vitro* antifungal activity against *Aspergillus*. Lamoth et al. (2015) reported that the *in vitro* activity of TSA against *A. fumigatus* was active with a MEC_50_, MIC_50_ and MIC_90_ were achieved at concentrations of 0.5, 1, and 4 μg/ml, respectively. Moreover, the combination of TSA with caspofungin was synergistic against *A. fumigatus.* TSA had little activity against *A. flavus* isolates (MEC_50_ > 16 μg/ml), but it had better activity against *A. niger*, *A. terreus*, *A. versicolor*, and *A. ustus* (MEC_50_ = 2 μg/ml for 90% of these isolates) (Lamoth et al., 2014, 2015).

Vorinostat (SAHA) is a novel HDAC inhibitor which is the analog of TSA that has a prolonged half-life and enhanced oral bioavailability. Tu et al. (2020) reported that the ranges of the MICs (100% inhibition) of SAHA against *Aspergillus* spp. were ≥ 16 μg/ml, when SAHA was combined with itraconazole (ITR), voriconazole (VRC) and posaconazole (POC), the synergistic effects were observed in *A. fumigatus* strains, *A. flavus* strains and *A. terreus* strains. The sessile minimal inhibitory concentrations (sMIC_80_) were defined as concentrations that reduced the biofilm’s metabolic activity by 80%. SAHA alone was ineffective against the biofilm, and sMIC_80_ was greater than 128 μg/ml. For the combination of SAHA and triazoles, the fractional inhibitory concentration index (FICI) revealed synergistic effects against *Aspergillus* spp. (Tu et al., 2020).

Givinostat is a class I and class II HDAC inhibitor that can be taken orally and has anti-inflammatory characteristics (Sun et al., 2017). All *Aspergillus* isolates examined by givinostat alone showed minimal antifungal efficacy. Favorable synergistic effects between triazoles and givinostat were shown against *A. fumigatus* and *A. flavus* (Sun et al., 2017).

MGCD290 is a novel Hos2 histone inhibitor. Pfaller et al. (2009) revealed that MGCD290 demonstrated synergy against *Aspergillus* spp. when it was combined with fluconazole, voriconazole and voriconazole. The triazoles changed to a more susceptible category with 70% (7 of 10) *Aspergillus* isolates when in combination with MGCD290 (Pfaller et al., 2009).

## Discussion and conclusion

Histone post-translational modifications play an important role in various cellular progress. In the past decade, advances in mass spectrometry-based proteomics provides new insights into the regulatory scope of post-translational modifications of non-histone proteins, which links to the regulation of pivotal pathways related to cellular response and protein functions (Buuh et al., 2017; Narita et al., 2019; Yang et al., 2022). SntB is a kind of epigenetic reader protein that assists in the recognition of histone tails. In *A. nidulans*, SntB was functioned as a transcriptional regulator that participated in the regulation of sterigmatocysin biosynthetic gene cluster (BGC). In *A. flavus*, SntB regulated H3K9 and H3K14 acetylation, aflatoxin synthesis, sclerotia formation and SM biosynthesis (Pfannenstiel et al., 2018). Cytological observations initially divided chromatin into two categories: euchromatin and heterochromatin (van Steensel, 2011). Heterochromatin protein 1 (HP1) can be involved in maintaining heterochromatin stability and transcriptional silencing, regulating gene expression, DNA replication repairmen (Leopold et al., 2019). In *A. nidulans*, hpeA is the homolog of HP1. Deletion of *hepA* resulted in the upregulation of SM biosynthesis genes, the genes involved in isopenicillin A production (*ipnA*) and in terraquinone A biosynthesis (*tdiB*) were significantly overexpressed (Reyes-Dominguez et al., 2010). In *A. oryzae*, SppA (signal peptide peptidase) is the homolog of *S. cerevisiae* SPP1. As a member of the COMPASS complex associated with Set1, SppA was involved in the SMs production including astellolides by affecting the methylation status of H3K4 (Shinohara et al., 2016).

In recent years, with the continuous development of medical treatments such as organ transplantation, immunosuppressants application, radiotherapy and chemotherapy, the incidence of invasive aspergillosis has increased. In addition, it has been reported that the resistance rate has increased. The increased morbidity and the limitation of therapeutic drugs prompted us to explore the mechanisms about the pathogenesis of *Aspergillus*. The discovery of novel antifungal targets and new antifungal agents is urgently needed. There are abundant histone posttranslational modifications in *Aspergillus*, possessing an important impact on protein structure, protein activity, stability, localization and protein-protein interactions, thereby regulating fungal growth, virulence, stress response and antifungal drug sensitivity in *Aspergillus*. In this article, we focused on several major *Aspergillus* (*A. fumigatus*, *A. flavus*, *A. niger*, *A. terreus*, *A. nidulans*, *A. oryzae*) and primary histone post-translational modifications including the process of histone acetylation, deacetylation, methylation, demethylation, and sumoylation (Zhao et al., 2011). More critically, we highlighted the essential role of HPTMs in fungal growth, pathogenicity and drug resistance, the potential anti-*Aspergillus* compounds targeting HPTMs were also summarized. This review will help us to further understand the relationship between HPTMs and pathogenicity in *Aspergillus*. Understanding the functions of HPTMs would contribute us to develop the fungal-specific targets, therefore probably reducing the side effects to the host. These findings will also contribute to the field of epigenetic regulation involved in the *Aspergillus* development and virulence. We believe that by learning more details between HPTMs and *Aspergillus*, it will make a lot of sense for developing new antifungal targets against fungal infections, as well as providing a theoretical foundation for fungus prevention and management.

## Author contributions

YL wrote the review and created the figures. All authors contributed significantly and intellectually to the work, and they all gave their consent for it to be published.
